# Comparison of Antimicrobial Resistance and Pan-Genome of Clinical and Non-Clinical *Enterococcus cecorum* from Poultry Using Whole-Genome Sequencing

**DOI:** 10.3390/foods9060686

**Published:** 2020-05-26

**Authors:** Poonam Sharma, Sushim K. Gupta, John B. Barrett, Lari M. Hiott, Tiffanie A. Woodley, Subhashinie Kariyawasam, Jonathan G. Frye, Charlene R. Jackson

**Affiliations:** 1 Bacterial Epidemiology and Antimicrobial Resistance Research Unit, United States Department of Agriculture, Agricultural Research Service, U.S. National Poultry Research Center, Athens, GA 30605, USA; poonamncl@gmail.com (P.S.); sushim.gupta@okstate.edu (S.K.G.); bennybarrett09@gmail.com (J.B.B.); lari.hiott@usda.gov (L.M.H.); tiffanie.woodley@usda.gov (T.A.W.); jonathan.frye@usda.gov (J.G.F.); 2 Department of Comparative, Diagnostic, and Population Medicine, College of Veterinary Medicine, The University of Florida, Gainesville, FL 32610, USA; skariyawasam@ufl.edu

**Keywords:** *Enterococcus*, genomics, antimicrobial resistance, poultry

## Abstract

*Enterococcus cecorum* is an emerging avian pathogen, particularly in chickens, but can be found in both diseased (clinical) and healthy (non-clinical) poultry. To better define differences between *E. cecorum* from the two groups, whole-genome sequencing (WGS) was used to identify and compare antimicrobial resistance genes as well as the pan-genome among the isolates. Eighteen strains selected from our previous study were subjected to WGS using Illumina MiSeq and comparatively analyzed. Assembled contigs were analyzed for resistance genes using ARG-ANNOT. Resistance to erythromycin was mediated by *ermB, ermG*, and *mefA*, in clinical isolates and *ermB* and *mefA,* in non-clinical isolates. Lincomycin resistance genes were identified as *linB*, *lnuB*, *lnuC*, and *lnuD* with *lnuD* found only in non-clinical *E. cecorum*; however, *lnuB* and *linB* were found in only one clinical isolate. For both groups of isolates, kanamycin resistance was mediated by *aph3-III*, while tetracycline resistance was conferred by *tet*M, *tet*O, and *tet*L. No mutations or known resistance genes were found for isolates resistant to either linezolid or chloramphenicol, suggesting possible new mechanisms of resistance to these drugs. A comparison of WGS results confirmed that non-clinical isolates contained more resistance genes than clinical isolates. The pan-genome of clinical and non-clinical isolates resulted in 3651 and 4950 gene families, respectively, whereas the core gene sets were comprised of 1559 and 1534 gene families in clinical and non-clinical isolates, respectively. Unique genes were found more frequently in non-clinical isolates than clinical. Phylogenetic analysis of the isolates and all the available complete and draft genomes showed no correlation between healthy and diseased poultry. Additional genomic comparison is required to elucidate genetic factors in *E. cecorum* that contribute to disease in poultry.

## 1. Introduction

*Enterococcus cecorum* has been implicated as a possible cause of disease in poultry, including spondylitis, vertebral osteoarthritis and femoral osteomyelitis. The bacterium normally resides as a member of the physiological microbiota of the intestinal tract of birds and mammals [1,2,3,4] and a dominant member of the enterococcal gastrointestinal microbiota of chickens [5]. It was first described as *Streptococcus cecorum* isolated from chickens in 1983 [6] and initially associated with clinical disease in poultry in Scotland in 2002 [7] followed by the Netherlands [8]. Since then, it has been reported as causing skeletal disease in broilers [9,10] and also in several outbreaks in Europe, [7,8,11,12,13,14,15,16] North America [17,18,19,20,21], Africa, [22], and eastern Asia [23].

As documented for many species within the *Enterococcus* genus, resistance to multiple classes of antimicrobials such as the aminoglycosides, *β*-lactams, macrolides, streptogramins, and tetracyclines have also been reported for *E. cecorum* [24]. These resistance phenotypes may partially explain why antimicrobial treatment has been ineffective in controlling mortality in poultry during outbreaks caused by *E. cecorum* [14]. In addition to antimicrobial resistance, genomic studies of *Enterococcus* in poultry are needed to compare the genetic composition of non-pathogenic and pathogenic *E. cecorum* strains to elucidate differences between the two groups. Presently there have been very few studies on comparison of resistance and genomes of *E. cecorum* from poultry [21,25] as well as comparison of non-pathogenic and pathogenic *E. cecorum* from different animal species [26] and chickens [25].

In our previous study, *E. cecorum* from diseased broiler chickens and poultry carcass rinsates were analyzed for antimicrobial resistance phenotype, virulence gene profile, and genetic relatedness [21].. Distinguishing the two groups of isolates was difficult based upon this phenotypic and genotypic characterization. The current study utilized comparative genomic analysis of selected *E. cecorum* isolates from diseased broiler chickens and poultry carcass rinsates to assess shared and unique genetic characteristics between the two groups. This data may aid in determining genetic relatedness and gene targets for the identification of pathogenic strains.

## 2. Materials and Methods

### 2.1. Bacterial Isolates and DNA Extraction

Isolates used in this analysis (*n* = 18) were selected from our previous study and included *E. cecorum* from non-clinical poultry collected by the animal arm of the National Antimicrobial Resistance Monitoring System (NARMS) from 2003–2011 and from diseased chickens from clinical cases submitted between 2008–2011 to the Animal Diagnostic Laboratory at the Pennsylvania State University [21]. *E. cecorum* genomic DNA was extracted using the blood and tissue genomic DNA extraction kit (Qiagen, Germantown, MD, USA). Extracted genomic DNA (gDNA) was quantified using Qubit double-stranded DNA (dsDNA) high-CHS) assay kit according to the manufacturer’s instructions (Life Technologies Inc., Carlsbad, CA, USA). The quality check of gDNA was performed using a NanoDrop™ spectrophotometer.

### 2.2. Sequencing, Assembly, and Annotation

*E. cecorum* sequencing libraries were prepared using a Nextera™ XT DNA Sample Preparation Kit and a Nextera™ XT Index Kit (Illumina Inc., San Diego, CA, USA). Illumina libraries were then quantified using a Qubit^®^ DNA HS Assay Kit in a Qubit fluorometer (Thermo Fisher Scientific, Waltham, MA, USA), and the size of the fragment libraries was checked using an Agilent 2100 Bioanalyzer System with an Agilent HS DNA Kit (Agilent Technologies, Santa Clara, CA, USA). Illumina libraries were sequenced on an Illumina MiSeq platform (Illumina Inc., San Diego, CA, USA) using a MiSeq v2 reagent kit with 500 cycles and a paired-end read length of 2 × 250 bp. Resulting paired-end sequencing reads were de novo assembled into contigs using A5-miseq assembler [27] and contigs were annotated using Prokka [28]. Prophage analysis was done using PHASTER [29]. The list of the web-based and command line tools used in our study is provided in Table S1.

### 2.3. Antimicrobial Susceptibility Testing and Prediction of Antimicrobial Resistance Genes

Antimicrobial susceptibility phenotypes were determined as previously described using microbroth dilution according to Clinical and Laboratory Standards Institute (CLSI) [21]. Antimicrobial resistance genes were predicted using ARG-ANNOT [30].

### 2.4. Pan-Genome Construction

The pan-genome of 18 *E. cecorum* isolates was constructed using the Prokka resultant general feature files 3 (.gff) as input file, and the families of homologous genes for *E. cecorum* were computed using Roary with identity cut-off of 95% [31]. The distribution of core (gene families commonly shared by all genomes), accessory (gene families not shared by all genomes), and unique (genes exclusive to one genome) genes were obtained from the Roary generated clusters of homologous gene groups. The *E. cecorum* genomes were functionally characterized using the Clusters of Orthologous Groups (COG) database [32]. For each *E. cecorum* genome, all the protein-coding sequences obtained from Prokka were subjected to reversed position specific blast (rps-blast) [33] against COG [32], and the rps-blast output was used to assign the COG function profile to the genes. Once the genes were assigned to the COGs, they were clustered into 20 of 25 functional categories, which were further grouped into four major classes, as described previously [34]. In brief, COG categories amino acid transport and metabolism (E), carbohydrate transport and metabolism (G), nucleotide transport and metabolism (F), energy production and conversion (C), coenzyme transport and metabolism (H), lipid transport and metabolism (I), inorganic ion transport and metabolism (P), and secondary metabolite biosynthesis, transport and catabolism (Q) were included in functional class Metabolism. COG categories cell wall/membrane/envelope biogenesis (M), cell motility (N), cell cycle control, cell division, chromosome partitioning (D), posttranslational modification, protein turnover, chaperones (O), signal transduction mechanisms (T), intracellular trafficking, secretion, and vesicular transport (U), and defense mechanisms (V) were included in functional class cellular processes and signaling. COG categories translation, ribosomal structure and biogenesis (J), transcription (K) and replication, recombination and repair (L) were included in functional class information storage, and processing and COG categories function unknown (S) and general function prediction only (R) were included in functional class poorly categorized. The core genes of clinical and non-clinical isolates were further categorized to different COG functional groups and compared with overall (clinical and non-clinical) core genes in different COG functional groups. The details of unique genes in all the clinical and non-clinical isolates is provided (Table S2).

### 2.5. Core-Genome Phylogenetic Tree

The phylogenetic tree was constructed using the core genes of 18 *E. cecorum* of our study, and available closed and draft genome sequences of *E. cecorum* retrieved from NCBI Gen-Bank. The retrieved genomes were annotated with Prokka for consistency. The Prokka generated .gff files were used as input in Roary to obtain core genome alignment. The core genome alignment was used for maximum likelihood (ML) phylogenetic tree generation using RAxML [35] with the general time-reversible (GTR) model of nucleotide evolution and gamma-distributed rate variation. FigTree (http://tree.bio.ed.ac.uk/software/figtree/) was used to graph the phylogenetic tree.

### 2.6. Nucleotide Accession Numbers

The *Enterococcus cecorum* genome sequences were deposited in GenBank under BioProject PRJNA580016 and accession numbers of WJEH00000000 to WJEY00000000 (Table S3).

## 3. Results

### 3.1. Genome Characteristics

Genome sizes of the 18 sequenced *E. cecorum* isolates ranged from 2.21 to 2.69 Mb, with 2120 to 2666 predicted coding genes and GC content from 36.0% to 36.6% (Table S3).

### 3.2. Antimicrobial Resistance Genes

Resistance genes reported were based on phenotypic results (Table 1). Differences in the antibiotic resistance patterns were noted between clinical and non-clinical isolates. Clinical isolates exhibited lower prevalence of resistance to antibiotics, with isolates displaying resistance to tetracycline (6/9), erythromycin (6/9), lincomycin (3/9), tylosin (2/9), kanamycin (1/9), and streptomycin (1/9). Alternatively, non-clinical isolates exhibited resistance to lincomycin (8/9) and tetracycline (8/9), followed by quinupristin–dalfopristin (Q/D; 7/9), tylosin (6/9), erythromycin (4/9), kanamycin (3/9), linezolid (3/9), and chloramphenicol (1/9). None of the clinical isolates were resistant to linezolid, Q/D, and chloramphenicol.

Clinical isolates showed the highest resistance to erythromycin, and erythromycin resistance genes *ermB* and/or *ermG* were, in some instances, found in conjunction with efflux pumps such as *mefA* and/or *msrD* in both clinical and non-clinical isolates (Table 1). These efflux pumps could also play a role in the resistance to lincomycin in both sets of isolates. Both *ermB* or *msrD* also conferred resistance to the streptogramin B component of Q/D and were found in seven Q/D resistant non-clinical isolates. Tetracycline resistant isolates harbored *tet*(L), *tet*(M), or *tet*(O) while kanamycin resistance was conferred by *aphIII* in both sets of isolates. Approximately 33% (3/9) of clinical isolates and 89% (8/9) of non-clinical isolates were resistant to lincomycin, and corresponding *lnu* and *lin* genes were found in all of those isolates (Table 1). Only one clinical isolate was resistant to streptomycin and one non-clinical isolate resistant to chloramphenicol; no known resistance mechanism was detected to either drug.

### 3.3. Pan-Genome Analysis

**Figure 1:**
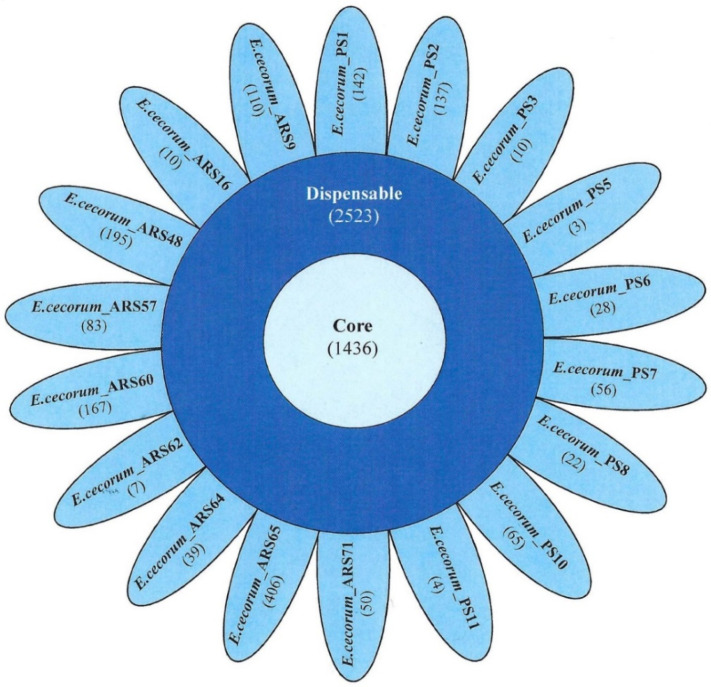
Venn diagram showing the pan-genome of 18 *Enterococcus cecorum* clinical (PS) and non-clinical (ARS) isolates from chicken. The number of core genes is the number of common genes shown in the center, while genes common between isolates are shown in the periphery (accessory genes). Each petal represents the unique genes in the respective isolates.

The pan-genome analysis for the *E. cecorum* isolates was initiated with 41,650 protein-coding sequences that resulted in 5493 gene families. The core gene set comprised 1436 genes, i.e., 62% of the average number of protein-coding sequences (2314 per genome), suggesting that more than half of the protein-coding sequences were part of the core and just over 1/3 of the protein-coding sequences in each genome were dispensable. The number of core, accessory, and unique genes in each genome is represented as a floral diagram in which the inner circle, outer circle, and petals represent core, accessory, and unique genes, respectively (Figure 1). The unique genes present in clinical isolates ranged from 3–142, whereas those in non-clinical isolates ranged from 7–406.

**Figure 2:**
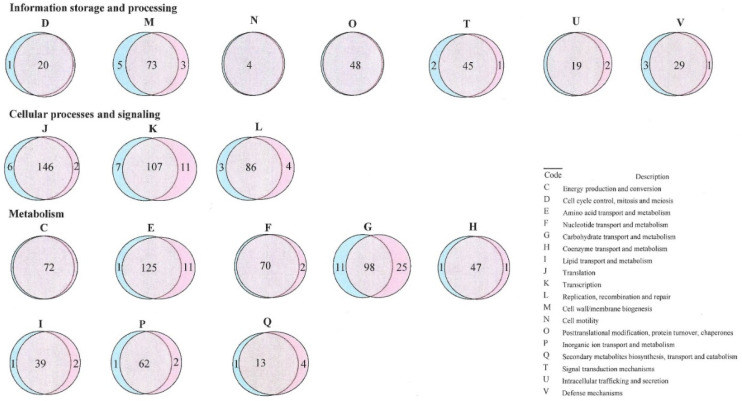
Venn diagram showing the Clusters of Orthologous Groups (COG) function categories of 18 *Enterococcus cecorum* clinical (PS; shown in pink) and non-clinical (ARS; shown in teal) isolates from chicken.

The comparison of core genes in different COG categories between clinical and non-clinical isolates is presented in the Venn diagram (Figure 2). Core genes of all of the *E. cecorum* isolates were assigned to 20 of 25 functional COG categories. Overall, the major class metabolism (CEFGHIPQ) was comprised of 36.63% of the core genes, while 23.61% and 16.57% of core genes were ascribed to class information storage and processing (JKL) and cellular process and signaling (DMNOTUV).

The core gene comparison of the isolates between clinical and non-clinical isolates revealed that the majority of genes were common; however, a few genes were more frequent in either clinical or non-clinical isolates. A high number of the core genes from functional class metabolism (CEFGHIPQ) and information storage and processing (JKL) were detected in the clinical isolates, while the gene number was higher for functional class cellular processes and signaling (DMNOTUV) in non-clinical isolates (Table S4). This revealed variation in the functional profile among *E. cecorum* isolates.

**Figure 3:**
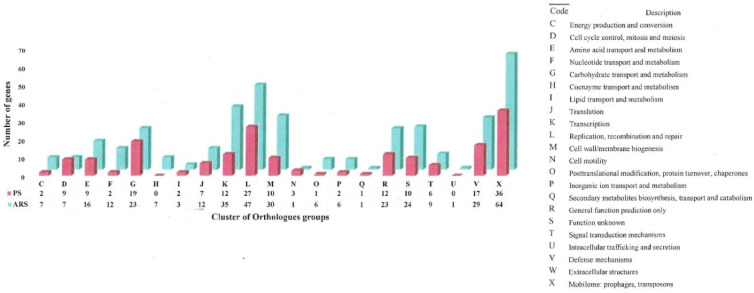
Distribution of unique genes in each Cluster of Orthologous Groups (COG) categories in clinical (PS; shown in pink) and non-clinical (ARS; shown in teal) *Enterococcus cecorum* isolates from chicken. The number of unique genes for each cluster is shown below the cluster code.

The unique genes were further assigned to different COG categories (Figure 3). Almost all of the COG categories were high in non-clinical isolates except for cell cycle control, mitosis and meiosis (D), and cell motility (N), which were slightly higher in clinical isolates. The most abundant COG category in both sets of isolates was mobilome: prophages, transposons (X). In the non-clinical isolates, the genes encoding for transcription (K), replication, recombination and repair (L), cell wall/membrane biogenesis (M), functions unknown (S), mobilome: prophages, transposons (X), and defense mechanisms (V) genes were significantly higher.

### 3.4. Core Genome Phylogenetic Analysis

**Figure 4:**
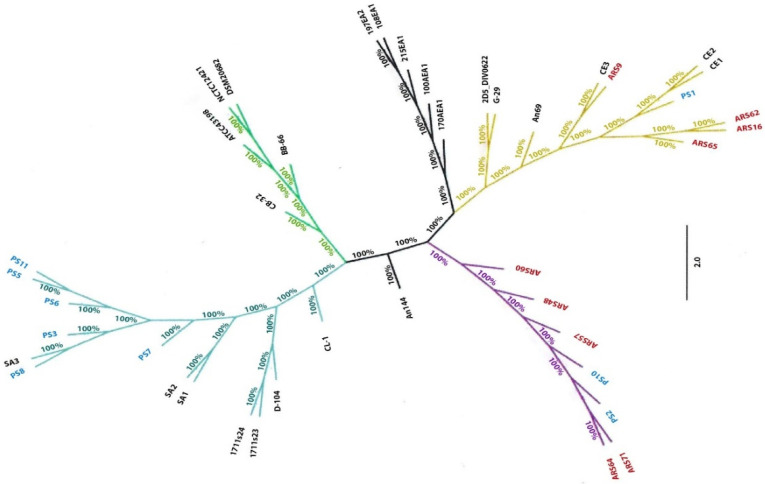
Core genome phylogenetic tree of 42 *Enterococcus cecorum* strains. The core genome phylogenetic tree was generated using the core-genome of the *E. cecorum* isolates from this study and 24 *E. cecorum* genomes from NCBI GenBank.

The core genome phylogenetic tree was generated using the core genome of the *E. cecorum* isolates from this study and 24 E. cecorum genomes from NCBI GenBank (Table S5). Some of the clinical isolates (PS3, PS5, PS6, PS7, PS8, and PS11) clustered with pathogenic reference isolates (SA1, SA2, and SA3) from chickens, while non-clinical (ARS9, ARS16, ARS62, and ARS65) isolates, including a single clinical isolate PS1, clustered with commensal reference isolates (CE1, CE2, and CE3) and formed a separate clade (Figure 4). Remaining clinical (PS2, PS10) and non-clinical isolates (ARS48, ARS57, ARS60, ARS64, ARS71) clustered together and branched out in the phylogenetic tree.

### 3.5. Prophage Analysis

The total number of intact prophages found in clinical and non-clinical isolates were nine and eight, respectively (Table 2). Intact prophages were not detected in all the isolates. Non-clinical isolate ARS65 had the highest number of intact prophages (four), whereas three clinical isolates (PS5, PS6, and PS11) had two each. There was no correlation between clinical and non-clinical isolates and the occurrence of intact prophages. The most frequent intact prophage detected was associated with *Streptococcus* genera. Three *Enterococcus* prophages were detected in non-clinical isolates only. The other prophages detected were associated with *Listeria* and *Bacillus* genera.

## 4. Discussion

In this study, eighteen *E. cecorum* were sequenced and compared based upon antibiotic resistance mechanisms and genome characteristics. The genome size and GC content of *E. cecorum* used in this study were similar to the generally observed genome sizes of other *Enterococcus* species. The average size of *E. cecorum* genomes was 2.38 Mb, and the average GC content was 36.35%. However, it was interesting to note that the average genome size of clinical isolates was less (2.33 Mb) than non-clinical isolates (2.44 Mb), which contradicts a previous study in which the size of commensal *E. cecorum* genomes were less than pathogenic genomes [25]. As a result, the average number of protein-coding sequences was also less in the clinical isolates (2264) as compared to non-clinical isolates (2363). The present study attempted to decipher the concordance between phenotypic resistance and genetic determinants. Results from this study correlated resistance determinants to their phenotypic expression in agreement with other studies on the use of WGS as a surveillance tool to detect antibiotic resistance [36,37]. Our results showed that non-clinical isolates exhibited resistance to more antibiotics and, thus, the presence of more antibiotic resistance genes in comparison to clinical isolates.

*E. cecorum* from poultry appear to be highly resistant to macrolides [24] and, in this study, erythromycin-resistance was mediated by either the presence of one or more resistance genes (*ermB, ermG),* and/or efflux genes (*mefA, msrD*) in both clinical and non-clinical isolates. The erythromycin resistance gene, *ermB*, is present in both humans and animal isolates and can be harbored on transposons and plasmids [38,39,40]. In the present study, *ermB* was associated on a plasmid in a clinical isolate or was likely to be harbored on a mobile genetic element in non-clinical isolates. The finding was similar to previous studies in which a higher prevalence of resistance to erythromycin was noticed in pathogenic isolates [19,41,42]. Genes conferring resistance to erythromycin and streptogramin B antibiotics such as *mefA* and *msrD*, respectively, and lincomycin resistance genes such as *linB*, *lnuB*, *lnuC*, and *lnuD* were identified. The *linB*, *lnuB* genes were common in both sets of isolates exhibiting resistance; however, two clinical *E. cecorum* isolates harbored *lnuC*, and only one non-clinical isolate contained *lnuD* along with other resistance genes. Upon further analysis of the contigs harboring lincomycin resistance genes, some of them were present on a plasmid in non-clinical isolates, but not in any of the clinical isolates where the genes were simply present in the genome.

Notably, three non-clinical *E. cecorum* isolates were simultaneously resistant to erythromycin, lincomycin, and Q/D, suggesting a possible acquired macrolide-lincosamide-streptogramin type B (MLS_B_) and streptogramin type A co-resistance. Although we have previously detected streptogramin type A resistance genes *vatB*, *vatD*, *vatE*, and *vgaB* from enterococci from poultry carcass rinsates [43,44], none of these genes were present in the *E. cecorum* in this study.

In both groups of isolates, kanamycin resistance was mediated by the aminoglycoside-modifying enzyme, *aph3-III,* and was associated with a plasmid in a single non-clinical isolate. Tetracycline resistance genes for ribosomal protection (*tet*(M), *tet*(O)) or *tet*(L) for efflux were detected in both sets of isolates. The *tet*(M) gene is most frequently found in *Enterococcus* [45] and, not surprising, overall resistance to tetracycline is linked to the use of this antibiotic as a therapeutic agent in poultry [42].

Phenotypic linezolid resistance in non-clinical isolates was not linked to any known mechanisms for resistance to linezolid, such as 23S rRNA gene mutation [46] or horizontally acquired *cfr* and *optrA* resistance genes [47,48]. However, linezolid resistance could also be due to cell wall thickness and biofilm formation [49] or overestimation of minimum inhibitory concentration [50]. Similarly, no mechanisms of resistance to chloramphenicol were detected in the one chloramphenicol resistant non-clinical isolate. Interestingly, this isolate was also resistant to linezolid. This could suggest possible new and/or alternative mechanisms of resistance to these antibiotics, which should be further examined.

The pan-genome of the 18 *E. cecorum* isolates comprised 41,650 protein-coding sequences, of which 1436 genes were conserved across all studied isolates. This finding is in concordance with the previous study on *E. faecalis* strains [51,52]. The pan-genome of clinical and non-clinical isolates resulted in 3651 and 4950 gene families, respectively, whereas the core gene sets were comprised of 1559 and 1534 gene families in clinical and non-clinical isolates, respectively. Comparison of the core genes of clinical and non-clinical isolates with the core genes of all the isolates revealed that there were 123 additional genes included in the core genes of clinical isolates, while 98 additional genes were included in the core genes of non-clinical isolates. The high number of accessory genes in non-clinical isolates compared to clinical isolates was attributed to a large number of accessory genes for maintaining replication and cell division that these commensal isolates possessed.

The pan-genome size increased when genomes of clinical and non-clinical isolates were assessed, which indicated an open pan-genome for the *E. cecorum* isolates and reduced core-genome size. The minute influence on the size of core genes with the addition of genomes indicated that the analyzed *E. cecorum* genomes were sufficient to construct the representative core-genome. The results were in agreement with previous studies of *E. faecalis* [53,54]. The small pan-genome size and inclusion of 43% gene families in core genes in the clinical isolates showed their conserved nature. Non-clinical isolates had large pan-genomes as compared to clinical isolates, and only 31% of gene families were part of the core genes and a higher number of unique genes. This data suggested that these isolates acquired a diverse set of genes to perform novel functions for better sustainability [55]. The distribution of the genes in different functional categories revealed variation in the functional profile among *E. cecorum* isolates. The functional analysis of core genes revealed that 6.82% (98/1436) of genes were ascribed to carbohydrate metabolism, which contrasted to an earlier report where 11.3% of core genes were ascribed to carbohydrate metabolism in *E. faecium* [54]. Moreover, about 7.88% (123/1559) and 7.11% (109/1534) of core genes were ascribed to carbohydrate metabolism in clinical and non-clinical isolates, respectively, which clearly indicates low intra-species and high inter-species metabolic gene variations in *Enterococcus* sp. The relative higher number of core genes were ascribed to carbohydrate metabolism in pathogenic isolates, and this indicates that these genes are essential to utilize various carbohydrates [56]. The relatively high number of genes in the core genome of the clinical isolates in class metabolism and class information storage and processing indicated that these genes are essential and conserved in clinical isolates. Alternatively, the relatively high number of genes in the core genome of the non-clinical isolates in class cellular processes and signaling showed that these genes are essential for better adaptability. A large number of genes were hypothetical genes which were assigned with unknown function. These genes may have additional functions associated with adaptability and survival, and this requires further research.

Among unique genes, high fractions (100/550) of genes were assigned to the mobilome group, and about 2/3 of this group of genes were in non-clinical isolates, suggesting that non-clinical isolates are contributing more to the population [57]. A unique sortase (surface protein transpeptidase) gene was present in clinical isolate PS8, and this surface protein may play a key role in the infection process [58]. Further studies will be required to examine the unexplored attributes.

Phylogenetic analysis of the core-genome of our 18 isolates along with core-genomes of 24 *E. cecorum* available in NCBI was performed. Some of the clinical and non-clinical isolates clustered, respectively, with previously reported pathogenic and commensal *E. cecorum* strains. However, no profound correlation between the strain origins to its phylogeny was established, which was in accordance with the inference drawn by earlier studies [59].

## 5. Conclusions

In conclusion, comparison of antimicrobial resistance and the genome of clinical and non-clinical *E. cecorum* isolates provided valuable insight about fundamental genetic differences observed between the two groups, which were different from previous findings on pathogenic and commensal *E. cecorum* isolates. The comparison of WGS results confirmed that non-clinical isolates contained more resistance genes than clinical isolates. Resistance genes were both shared and exclusive to each group indicating varying genetic characteristics among *E. cecorum* isolates. The pan-genome analysis revealed that the non-clinical genomes were comparatively diverse due to the acquisition of additional genes, while genome reduction in the conserved clinical genomes suggested better host adaptability. Further comparative investigation of additional genomes of non-clinical and clinical *E. cecorum* isolates may reveal attributes in clinical *E. cecorum* responsible for their pathogenic nature in poultry.

## Figures and Tables

**Table 1:** Antibiotic resistance profile of clinical and non-clinical *Enterococcus cecorum* isolates.

Isolate No.	Resistance Profile	Antibiotics Resistance Genes
PS1	Lincomycin-Tetracycline	*lnuB, linB, tetL, tetM, tetO*
PS2	Erythromycin-Kanamycin-Lincomycin-Tylosin	*ermG, ermB, mefA, msrD, aph3-III, lnuC*
PS3	Erythromycin-Streptomycin-Tetracycline	*ermG, mefA, msrD, tetL, tetM*
PS5	Erythromycin-Tylosin-Tetracycline	*ermG, mefA, msrD, tetL, tetM*
PS6	Erythromycin-Tetracycline	*ermG, mefA, msrD, tetL, tetM*
PS7	Lincomycin	*lnuC*
PS8	Erythromycin-Streptomycin-Tetracycline	*ermG, mefA, msrD, tetL, tetM*
PS11	Erythromycin-Tetracycline	*ermG, mefA, msrD, tetL, tetM*
ARS9	Linezolid-Quinupristin/Dalfopristin	*ermB*
ARS16	Erythromycin-Kanamycin-Lincomycin-Quinupristin/Dalfopristin-Tetracycline	*mefA, msrD, aph3-III, lnuB, linB, tetL, tetM, tetO*
ARS48	Erythromycin-Lincomycin-Quinupristin/Dalfopristin-Tylosin-Tetracycline	*ermB, mefA, msrD, lnuB, linB, tetM*
ARS57	Lincomycin-Linezolid-Quinupristin/Dalfopristin-Tylosin-Tetracycline	*ermB, mefA, msrD, lnuB, linB, lnuD*
ARS60	Kanamycin-Lincomycin-Quinupristin/Dalfopristin-Tetracycline	*ermB, msrD, aph3-III, lnuB, linB, tetL, tetM*
ARS62	Kanamycin-Lincomycin-Quinupristin/Dalfopristin-Tylosin-Tetracycline	*ermB, msrD, aph3-III, lnuB, linB, tetL, tetM, tetO*
ARS64	Erythromycin-Lincomycin-Tylosin-Tetracycline	*ermB, tetL, tetM*
ARS65	Chloramphenicol-Lincomycin-Linezolid-Quinupristin/Dalfopristin-Tylosin-Tetracycline	*ermB, mefA, msrD, lnuB, linB, tetL, tetM, tetO*
ARS71	Erythromycin-Lincomycin-Tylosin-Tetracycline	*ermB, tetL, tetM*

**Table 2:** List of intact phage in *Enterococcus cecorum* isolates from healthy and diseased chickens.

Isolate	Length	GC	CDS	Best Match	
				Phage	Accession Number
ARS16	36.3	37.17	47	PHAGE_Strept_5093	NC_012753
ARS48	31.1	37.87	43	PHAGE_Entero_EFC_1	NC_025453
ARS57	47.3	37.59	52	PHAGE_Entero_EFC_1	NC_025453
ARS62	36.3	37.17	47	PHAGE_Strept_5093	NC_012753
ARS64	36.1	35.41	43	PHAGE_Lister_vB_LmoS_188	NC_028871
ARS65	33.6	37.60	43	PHAGE_Entero_EFC_1	NC_025453
	34.4	36.85	23	PHAGE_Bacter_Diva	NC_028788
	14.7	39.26	17	PHAGE_Strept_5093	NC_012753
	14.5	38.74	16	PHAGE_Strept_5093	NC_012753
PS2	23.9	37.30	35	PHAGE_Strept_5093	NC_012753
PS5	30.7	35.37	36	PHAGE_Bacill_phBC6A52	NC_004821
	35.8	36.8	47	PHAGE_Strept_5093	NC_012753
PS6	30.7	35.37	36	PHAGE_Bacill_phBC6A52	NC_004821
	35.8	36.8	47	PHAGE_Strept_5093	NC_012753
PS8	32.2	36.34	39	PHAGE_Strept_5093	NC_012753
PS11	30.7	35.37	36	PHAGE_Bacill_phBC6A52	NC_004821
	35.8	36.8	47	PHAGE_Strept_5093	NC_012753

## Supplementary Materials

The following are available online at https://www.mdpi.com/2304-8158/9/6/686/s1. Table S1: List of online tools used in this study; Table S2: Clusters of Orthologous Groups (COG) in the clinical and non-clinical *Enterococcus cecorum* isolates; Table S3: Genome assembly statistics and metadata of clinical and non-clinical *Enterococcus cecorum*; Table S4: List of core gene predominantly present in *Enterococcus cecorum* isolates from healthy and diseased chickens; Table S5: List of *Enterococcus cecorum* genomes used for phylogenetic analysis.
