# Supplementary material for: Comparison of Antimicrobial Resistance and Pan-Genome of Clinical and Non-Clinical Enterococcus cecorum from Poultry Using Whole-Genome Sequencing
Source: Foods. 2020 May 26;9(6):686. doi: 10.3390/foods9060686 (PMC7353540; doi:10.3390/foods9060686)
Supplement: Supplementary file 1 [file foods-09-00686-s001.zip › supplementary/Supplementary Tables S3-S5.docx]

Table S3. Genome assembly statistics and metadata of clinical and non-clinical *Enterococcus cecorum*.

| **Isolate Name** | **Isolation source†** | **Year of Isolation** | **Place of isolation‡** | **Clinical/**  **Non-clinical*** | **Contigs** | **Genome size (bp)** | **N50** | **CDS number** | **Genome coverage** | **% GC** | **NCBI accession number** |
| --- | --- | --- | --- | --- | --- | --- | --- | --- | --- | --- | --- |
| *E. cecorum*_PS1 | D | 2009 | PSU | C | 109 | 2454530 | 58414 | 2345 | 77x | 36.3 | WJEY00000000 |
| *E. cecorum*_PS2 | D | 2009 | PSU | C | 112 | 2481576 | 52859 | 2411 | 70x | 36.2 | WJEX00000000 |
| *E. cecorum*_PS3 | D | 2009 | PSU | C | 54 | 2220794 | 114003 | 2144 | 119x | 36.4 | WJEW00000000 |
| *E. cecorum*_PS5 | D | 2008 | PSU | C | 68 | 2245430 | 92885 | 2191 | 109x | 36.6 | WJEV00000000 |
| *E. cecorum*_PS6 | D | 2008 | PSU | C | 67 | 2272393 | 102731 | 2219 | 89x | 36.5 | WJEU00000000 |
| *E. cecorum*_PS7 | D | 2008 | PSU | C | 74 | 2386877 | 77631 | 2314 | 98x | 36.4 | WJET00000000 |
| *E. cecorum*_PS8 | D | 2008 | PSU | C | 55 | 2269443 | 131502 | 2206 | 112x | 36.4 | WJES00000000 |
| *E. cecorum*_PS10 | D | 2008 | PSU | C | 108 | 2460755 | 56896 | 2356 | 113x | 36 | WJER00000000 |
| *E. cecorum*_PS11 | D | 2009 | PSU | C | 65 | 2243031 | 102731 | 2192 | 116x | 36.6 | WJEQ00000000 |
| *E. cecorum*_ARS9 | CC | 2006 | NARMS | NC | 118 | 2347259 | 55338 | 2263 | 114x | 36.3 | WJEP00000000 |
| *E. cecorum*_ARS16 | CC | 2007 | NARMS | NC | 125 | 2456889 | 47963 | 2332 | 86x | 36.3 | WJEO00000000 |
| *E. cecorum*_ARS48 | CC | 2003 | NARMS | NC | 91 | 2477197 | 82914 | 2446 | 114x | 36.5 | WJEN00000000 |
| *E. cecorum*_ARS57 | CC | 2009 | NARMS | NC | 86 | 2301548 | 77696 | 2212 | 116x | 36.4 | WJEM00000000 |
| *E. cecorum*_ARS60 | CC | 2004 | NARMS | NC | 121 | 2670699 | 59682 | 2648 | 72x | 36.1 | WJEL00000000 |
| *E. cecorum*_ARS62 | CC | 2004 | NARMS | NC | 121 | 2454653 | 58535 | 2336 | 73x | 36.3 | WJEK00000000 |
| *E. cecorum*_ARS64 | CC | 2010 | NARMS | NC | 66 | 2216296 | 67333 | 2120 | 113x | 36.6 | WJEJ00000000 |
| *E. cecorum*_ARS65 | CC | 2010 | NARMS | NC | 94 | 2698166 | 81800 | 2666 | 74x | 36.1 | WJEI00000000 |
| *E. cecorum*_ARS71 | CC | 2010 | NARMS | NC | 77 | 2351405 | 60711 | 2249 | 93x | 36.3 | WJEH00000000 |

**†**CC = Chicken carcass; D = Diseased **‡**NARMS = National Antimicrobial Resistance Monitoring System; PSU = Pennsylvania State University, PA *NC = Non-Clinical; C = Clinical

Table S4. List of core genes predominantly present in *Enterococcus cecorum* isolates from healthy and diseased chicken.

| **Clinical isolate (PS)** | | |
| --- | --- | --- |
| **Functional categories** | **COG protein description** | **COG ID** |
| E | Dihydrodipicolinate synthase/N-acetylneuraminate lyase | COG0329 |
|  | Arginase/agmatinase/formimionoglutamate hydrolase, arginase family | COG0010 |
|  | Histidinol-phosphate/aromatic aminotransferase and cobyric acid decarboxylase | COG0079 |
|  | Imidazoleglycerol-phosphate dehydratase | COG0131 |
|  | Glutamine amidotransferase | COG0118 |
|  | Phosphoribosylformimino-5-aminoimidazole carboxamide ribonucleotide (ProFAR) isomerase | COG0106 |
|  | Imidazoleglycerol-phosphate synthase | COG0107 |
|  | Threonine aldolase | COG2008 |
|  | ABC-type proline/glycine betaine transport systems, ATPase components | COG1125 |
|  | Na+/serine symporter | COG3633 |
|  | ABC-type oligopeptide transport system, periplasmic component | COG4166 |
| F | ADP-ribose pyrophosphatase | COG1051 |
|  | ADP-ribose pyrophosphatase | COG1051 |
| G | Beta-glucosidase-related glycosidases | COG1472 |
|  | D-mannonate dehydratase | COG1312 |
|  | Glucuronate isomerase | COG1904 |
|  | Beta-galactosidase/beta-glucuronidase | COG3250 |
|  | Na+/melibiose symporter and related transporters | COG2211 |
|  | Putative N-acetylmannosamine-6-phosphate epimerase | COG3010 |
|  | Neuraminidase (sialidase) | COG4409 |
|  | ABC-type sugar transport system, permease component | COG0395 |
|  | ABC-type sugar transport systems, permease components | COG1175 |
|  | ABC-type sugar transport system, periplasmic component | COG1653 |
|  | Beta-galactosidase, beta subunit | COG2731 |
|  | Phosphotransferase system, mannose/fructose/N-acetylgalactosamine-specific component IIC | COG3715 |
|  | Phosphotransferase system, mannose/fructose/N-acetylgalactosamine-specific component IID | COG3716 |
|  | Phosphotransferase system, mannose/fructose/N-acetylgalactosamine-specific component IIC | COG3715 |
|  | Phosphotransferase system, mannose/fructose/N-acetylgalactosamine-specific component IIB | COG3444 |
|  | 5-keto 4-deoxyuronate isomerase | COG3717 |
|  | Phosphotransferase system cellobiose-specific component IIA | COG1447 |
|  | Phosphotransferase system, mannitol-specific IIBC component | COG2213 |
|  | Phosphomannose isomerase | COG1482 |
|  | Fructose-2,6-bisphosphatase | COG0406 |
|  | Sugar kinases, ribokinase family | COG0524 |
|  | Phosphotransferase system sorbitol-specific component IIA | COG3731 |
|  | 3-carboxymuconate cyclase | COG2706 |
|  | Phosphotransferase system cellobiose-specific component IIB | COG1440 |
| H | Lipoate-protein ligase A | COG0095 |
| I | Dehydrogenases with different specificities (related to short-chain alcohol dehydrogenases) | COG1028 |
|  | Dehydrogenases with different specificities (related to short-chain alcohol dehydrogenases) | COG1028 |
| J | Acetyltransferases, including N-acetylases of ribosomal proteins | COG1670 |
|  | Acetyltransferases, including N-acetylases of ribosomal proteins | COG1670 |
|  | Ribosomal protein L32 | COG0333 |
|  | Acetyltransferases, including N-acetylases of ribosomal proteins | COG1670 |
|  | 16S RNA G1207 methylase RsmC | COG2813 |
|  | tRNA nucleotidyltransferase/poly(A) polymerase | COG0617 |
| K | Transcriptional regulator | COG1309 |
|  | Transcriptional regulators | COG2186 |
|  | Transcriptional regulator/sugar kinase | COG1940 |
|  | Transcriptional regulators | COG1737 |
|  | Arginine repressor | COG1438 |
|  | Predicted transcriptional regulators | COG1476 |
|  | Transcriptional regulators | COG2188 |
|  | Transcriptional regulators | COG1609 |
|  | Response regulator of citrate/malate metabolism | COG4565 |
|  | Transcriptional regulators | COG1609 |
|  | Predicted transcriptional regulators | COG0789 |
| L | DNA-damage-inducible protein J | COG3077 |
|  | Predicted DNA alkylation repair enzyme | COG4912 |
|  | DNA-damage-inducible protein J | COG3077 |
|  | Transposase and inactivated derivatives | COG3436 |
| M | Phosphoglycerol transferase and related proteins, alkaline phosphatase superfamily | COG1368 |
|  | Periplasmic glycine betaine/choline-binding (lipo)protein of an ABC-type transport system (osmoprotectant binding protein) | COG1732 |
|  | Dihydrodipicolinate synthase/N-acetylneuraminate lyase | COG0329 |
| P | Chromate transport protein ChrA | COG2059 |
|  | Rhodanese-related sulfurtransferase | COG0607 |
| Q | Uncharacterized enzyme involved in pigment biosynthesis | COG2313 |
|  | Uncharacterized protein, possibly involved in aromatic compounds catabolism | COG2050 |
|  | Dehydrogenases with different specificities (related to short-chain alcohol dehydrogenases) | COG1028 |
|  | Dehydrogenases with different specificities (related to short-chain alcohol dehydrogenases) | COG1028 |
| R | Predicted metal-dependent membrane protease | COG1266 |
|  | Predicted phosphatases | COG0546 |
|  | Predicted dehydrogenases and related proteins | COG0673 |
|  | Predicted double-stranded RNA/RNA-DNA hybrid binding protein | COG3341 |
|  | Acetyltransferases | COG0456 |
|  | Predicted extracellular nuclease | COG2374 |
|  | Plasmid stabilization system protein | COG3668 |
|  | Predicted permease | COG0628 |
|  | Uncharacterized membrane protein, possible Na+ channel or pump | COG1811 |
|  | Predicted SAM-dependent methyltransferases | COG1092 |
|  | Aldo/keto reductases, related to diketogulonate reductase | COG0656 |
|  | Dehydrogenases with different specificities (related to short-chain alcohol dehydrogenases) | COG1028 |
|  | Dehydrogenases with different specificities (related to short-chain alcohol dehydrogenases) | COG1028 |
| S | Predicted membrane protein | COG1511 |
|  | Uncharacterized protein conserved in bacteria | COG4475 |
|  | Uncharacterized conserved protein | COG3832 |
|  | Uncharacterized conserved protein | COG5582 |
|  | Predicted membrane protein | COG3759 |
| T | Response regulator of citrate/malate metabolism | COG4565 |
| U | Preprotein translocase subunit YajC | COG1862 |
|  | Preprotein translocase subunit SecE | COG0690 |
| V | ABC-type multidrug transport system, ATPase component | COG1131 |
| **Non-clinical isolates (ARS)** | | |
| **Functional categories** | **COG protein description** | **COG ID** |
| D | Protein required for the initiation of cell division | COG4839 |
| E | Threonine aldolase | COG2008 |
| G | Beta-fructosidases (levanase/invertase) | COG1621 |
|  | ABC-type sugar transport system, periplasmic component | COG1653 |
|  | ABC-type sugar transport system, permease component | COG0395 |
|  | ABC-type polysaccharide transport system, permease component | COG4209 |
|  | Phosphotransferase system cellobiose-specific component IIB | COG1440 |
|  | Phosphotransferase system cellobiose-specific component IIA | COG1447 |
|  | Fructose-2,6-bisphosphatase | COG0406 |
|  | Beta-glucosidase/6-phospho-beta-glucosidase/beta-galactosidase | COG2723 |
|  | Phosphotransferase system cellobiose-specific component IIC | COG1455 |
|  | Cellulase M and related proteins | COG1363 |
|  | Transcriptional regulator/sugar kinase | COG1940 |
| H | GTP cyclohydrolase I | COG0302 |
| I | Dehydrogenases with different specificities (related to short-chain alcohol dehydrogenases) | COG1028 |
| J | Acetyltransferases, including N-acetylases of ribosomal proteins | COG1670 |
|  | Dimethyladenosine transferase (rRNA methylation) | COG0030 |
| K | Transcriptional regulator | COG1309 |
|  | Transcriptional regulators | COG1609 |
|  | Transcriptional regulator/sugar kinase | COG1940 |
|  | Transcriptional regulators | COG1737 |
|  | Transcriptional regulators | COG1737 |
|  | Transcriptional regulator | COG1309 |
| L | DNA-damage-inducible protein J | COG3077 |
|  | Transposase and inactivated derivatives | COG3464 |
|  | Transposase and inactivated derivatives | COG2801 |
|  | Guanosine polyphosphate pyrophosphohydrolases/synthetases | COG0317 |
| M | D-alanyl-D-alanine carboxypeptidase | COG1686 |
|  | Cell division septal protein | COG1589 |
|  | Sortase and related acyltransferases | COG1247 |
|  | Putative exporter of polyketide antibiotics | COG3559 |
|  | Putative exporter of polyketide antibiotics | COG3559 |
| P | ABC-type cobalt transport system, permease component CbiQ and related transporters | COG0619 |
| Q | Dehydrogenases with different specificities (related to short-chain alcohol dehydrogenases) | COG1028 |
| R | Predicted CoA-binding protein | COG1832 |
|  | Metal-dependent hydrolase | COG3568 |
|  | Acetyltransferases | COG0456 |
|  | Acetyltransferases | COG0456 |
|  | ABC-type transport system involved in multi-copper enzyme maturation, permease component | COG1277 |
|  | Dehydrogenases with different specificities (related to short-chain alcohol dehydrogenases) | COG1028 |
| S | Uncharacterized protein conserved in bacteria | COG1799 |
|  | Predicted membrane protein | COG4720 |
|  | Uncharacterized protein conserved in bacteria | COG4185 |
| T | Guanosine polyphosphate pyrophosphohydrolases/synthetases | COG0317 |
|  | Signal transduction histidine kinase | COG5002 |
| V | ABC-type multidrug transport system, ATPase component | COG1131 |
|  | ABC-type multidrug transport system, ATPase component | COG1131 |
|  | Uncharacterized bacitracin resistance protein | COG1968 |

Table S5. List of *Enterococcus cecorum* genomes used for phylogenetic analysis

| Organism/Name | Strain | Source | BioSample | Size (Mb) | GC% | Accession Number | Genome |
| --- | --- | --- | --- | --- | --- | --- | --- |
| *Enterococcus cecorum* | SA3 | Spinal lesions | SAMN03203014 | 2.32 | 36.4 | CP010064.1 | Complete |
| *Enterococcus cecorum* | SA2 | Spinal lesion | SAMN03203013 | 2.28 | 36.6 | CP010061.1 | Complete |
| *Enterococcus cecorum* | SA1 | Spinal lesion | SAMN03203012 | 2.26 | 36.6 | CP010060.1 | Complete |
| *Enterococcus cecorum* | CE3 | Ceca | SAMN03202987 | 2.37 | 36.2 | CP010063.1 | Complete |
| *Enterococcus cecorum* | CE2 | Ceca | SAMN03202986 | 2.43 | 36.3 | CP010062.1 | Complete |
| *Enterococcus cecorum* | CE1 | Ceca | SAMN03202984 | 2.43 | 36.3 | CP010059.1 | Complete |
| *Enterococcus cecorum* | NCTC12421 | Caeca | SAMEA3529272 | 2.42 | 36.6 | LS483306.1 | Complete |
| *Enterococcus cecorum* DSM 20682 = ATCC 43198 | ATCC 43198 | NA | SAMN02596960 | 2.40 | 36.5 | ASWI01 | Draft |
| *Enterococcus cecorum* DSM 20682 = ATCC 43198 | DSM 20682 | ceca | SAMN02256445 | 2.34 | 36.4 | ARBO01 | Draft |
| *Enterococcus cecorum* | 1711s24 | Cecal content | SAMN03702600 | 2.30 | 36.2 | LDOX01 | Draft |
| *Enterococcus cecorum* | 1710s23 | Cecal content | SAMN03702599 | 2.25 | 36.3 | LDOW01 | Draft |
| *Enterococcus cecorum* | 170AEA1 | Chicken Breast meat | SAMN02729151 | 2.40 | 36.7 | LEOY01 | Draft |
| *Enterococcus cecorum* | D-104 | Duck heart, liver | SAMN03699897 | 2.27 | 36.3 | LDDZ01 | Draft |
| *Enterococcus cecorum* | 215EA1 | Breast meat | SAMN02729164 | 2.20 | 36.7 | LEPA01 | Draft |
| *Enterococcus cecorum* | 108EA1 | Breast meat | SAMN02729141 | 2.23 | 36.7 | LEOX01 | Draft |
| *Enterococcus cecorum* | G-29 | Goose heart, liver | SAMN03699964 | 2.13 | 36.9 | LDEC01 | Draft |
| *Enterococcus cecorum* | CL-1 | Chicken | SAMN03699967 | 2.33 | 36.3 | LDEA01 | Draft |
| *Enterococcus cecorum* | An144 | Caecum | SAMN06473616 | 2.49 | 36.1 | NFLC01 | Draft |
| *Enterococcus cecorum* | An69 | Caecum | SAMN06473747 | 1.99 | 36.6 | NFHQ01 | Draft |
| *Enterococcus cecorum* | 197EA2 | Breast meat | SAMN02729159 | 2.30 | 36.9 | LEOZ01 | Draft |
| *Enterococcus cecorum* | 2D5_DIV0622 | Bird | SAMN04634068 | 2.47 | 37.1 | NIBL01 | Draft |
| *Enterococcus cecorum* | BB-66 | Broiler breeder chicken | SAMN03699965 | 2.48 | 36.2 | LDED01 | Draft |
| *Enterococcus cecorum* | CB-32 | Broiler chicken | SAMN03699966 | 2.29 | 36.5 | LDEB01 | Draft |
| *Enterococcus cecorum* | 100AEA1 | Breast meat | SAMN02729139 | 2.28 | 37.1 | LEOW01 | Draft |
