# Supplementary material for: Comparison of Antimicrobial Resistance and Pan-Genome of Clinical and Non-Clinical Enterococcus cecorum from Poultry Using Whole-Genome Sequencing
Source: Foods. 2020 May 26;9(6):686. doi: 10.3390/foods9060686 (PMC7353540; doi:10.3390/foods9060686)
Supplement: Supplementary file 1 [file foods-09-00686-s001.zip › supplementary/Table S1.docx]

Table S1: List of the online tools used in this study

| **Name** | **Description** | **Interface** | **Input** | **Source** |
| --- | --- | --- | --- | --- |
| **A5-miseq** | A pipeline for assembling reads generated on the Illumina sequencing platform. | Command line | Reads generated from Illumina platform | <https://sourceforge.net/projects/ngopt/> |
| **PROKKA** | A command line tool to annotate bacterial genome | Command line | Assembled genomes | <https://github.com/tseemann/prokka> |
| **PHASTER** | A web server for the rapid detection and annotation of prophage within bacterial genomes and plasmids | Web based | Assembled genomes | <https://phaster.ca/> |
| **Roary** | A high speed standalone pan genome pipeline, which takes annotated assemblies in gff3 format (generated by PROKKA) and calculates the pan genome | Command line | Features file (gff) | <https://sanger-pathogens.github.io/Roary/> |
| **RAxML** | A tool for Maximum-likelihood based phylogenetic inference | Command line | Alignment file (phylip) or fasta) | <https://cme.h-its.org/exelixis/web/software/raxml/index.html> |
| **FigTree** | A tool for graphical viewer of phylogenetic trees | Standalone | Newick | <http://tree.bio.ed.ac.uk/software/figtree/> |
| **ARG-ANNOT** | A standalone tool to detect antibiotic resistance genes in bacterial genomes | Standalone | Assembled genomes | <http://backup.mediterranee-infection.com/article.php?laref=282&titre=arg-annot> |
